# c.3623G > A mutation encodes a CFTR protein with impaired channel function

**DOI:** 10.1186/s12931-016-0326-7

**Published:** 2016-01-22

**Authors:** Xiaoying Zhang, Jaspal S. Hothi, Yanhui H. Zhang, Saumini Srinivasan, Dennis C. Stokes, Weiqiang Zhang

**Affiliations:** Department of Pediatrics, University of Tennessee Health Science Center, 50 North Dunlap Street, Memphis, TN 38103 USA; Department of Bioscience Research, University of Tennessee Health Science Center, 875 Union Avenue, Memphis, TN 38163 USA; Department of Physiology, University of Tennessee Health Science Center, 50 North Dunlap Street, Room 309R, Memphis, TN 38103 USA; University of Tennessee Cystic Fibrosis Care and Research Center, Le Bonheur Children’s Hospital-Methodist University Hospital, Memphis, TN 38103 USA

**Keywords:** Cystic fibrosis (CF), CFTR, CFTR mutations, CFTR modulators, VX-809

## Abstract

**Background:**

The aims of this study were to characterize clinical features of a pediatric African-American cystic fibrosis (CF) patient heterozygous for F508del and a novel c.3623G > A mutation, and to identify the molecular defect(s) associated with c.3623G > A mutation.

**Methods:**

The medical record of this patient was analyzed retrospectively. Western blotting and iodide efflux assay were used to study mutant CFTR protein expression level, maturation status, channel function, and the effects of CFTR modulation on these characteristics.

**Results:**

The encoding protein of c.3623G > A mutation, G1208D-CFTR, has a moderate processing defect and exhibits impaired channel function, which were partially rescued by using VX-809 or exposed to low temperature (28 °C). The patient has mild CF disease manifestations.

**Conclusions:**

Our biochemical findings correlate with the clinical phenotype and suggest that c.3623G > A is a CF-causing mutation. The study helps expand our knowledge of rare CFTR mutations in a minority population and may have important clinical implications for personalized therapeutic intervention.

## Background

Cystic fibrosis (CF) is an autosomal recessive genetic disease caused by the loss or dysfunction of the CF transmembrane conductance regulator (CFTR) channel activity resulting from mutations [1, 2]. Clinically, chronic lung disease is the main cause of morbidity and mortality for CF patients. Other symptoms include pancreatic insufficiency, elevated sweat electrolytes, gastrointestinal disorders, reproductive abnormalities, etc. [3, 4].

CFTR is a cAMP/cGMP-regulated chloride /bicarbonate channel primarily expressed at the apical membrane of epithelial cells lining airway, gut, and exocrine glands, where it is responsible for transepithelial fluid secretion and homeostasis [5–7]. CFTR channel function is determined by the number of channels at the cell surface, the open probability of the channels, and the single channel conductance. Mutations in the *CFTR* gene alter one or more of these parameters, causing the impairment or loss of the channel activity. More than 2000 CFTR mutations have been identified, which can be roughly categorized into 5 groups based on the nature of defect(s) [8]. The classification of CFTR mutations helps define strategies to restore CFTR channel function based on mutation-specific defect(s). It should be noted that some mutations have multiple defects. For example, F508del, the most prevalent CFTR mutation, induces a maturation defect in CFTR protein; when this maturation defect is rescued, the mutant protein still exhibits defects in channel gating and stability at the cell surface. Among the 2000 plus known CFTR mutations, only a relatively few have been studied in detail both at the molecular level and for the specific disease manifestations. In order to better understand the disease of CF and develop effective therapies, there is a need to study the molecular characteristics of rare CFTR mutations to identify the defect(s), particularly for rare mutations seen in minority populations such as African-Americans.

In addition to therapies targeting the downstream disease consequences (symptoms), recent advancements to target the mutant CFTR proteins (CFTR modulation) have potentially revolutionized CF care. Kalydeco™ (also known as ivacaftor or VX-770) was approved by U.S. Food and Drug Administration (FDA) to treat CF patients age 2 or older with G551D and other nine class III and IV mutations [9]. More recently, FDA approved Orkambi™ (a combination of ivacaftor and lumacaftor (also known as VX-809)) to treat CF patients age 12 or older with two copies of F508del [9].

Here, we present a clinical case of a pediatric African-American CF patient who is heterogeneous for F508del and a novel missense mutation, c.3623G > A. The patient had mild disease manifestations. Search in the available databases [10–12] did not yield any information on this mutation. The goals of this study were to characterize this novel mutation at the molecular level to identify the nature of defect(s), and to explore the possibility of using mutation-specific therapy for potential interventions.

## Methods

### Patient characteristics

This individual received standard care at The University of Tennessee Cystic Fibrosis Research and Care Center at LeBonheur Children’s Hospital (Memphis, TN, USA). The medical record was analyzed retrospectively after expedited IRB approval (UTHSC 13-02779-XM).

Genotyping was performed at Ambry Genetics (Aliso Viejo, CA, USA) that showed F508del mutation on one chromosome and c.3623G > A on the other.

Diagnostic sweat chloride testing was performed on the patient by using pilocarpine iontophoresis for duplicate samples from right to left arms. Collection was performed using the filter paper method according to CF Foundation/NCCLS guidelines [13]. The chloride concentrations were measured by using a digital chloridometer (Labconco, Kansas City, MO, USA) with a minimal sweat weight of 75 mg.

### Antibodies and reagents

Antibodies were purchased from the following companies: Anti-CFTR, clone MM13-4 (EMD Millipore Corporation, CA, USA), anti-β-Actin (Sigma, MO, USA). VX-809 was purchased from Selleckchem (TX, USA). Other reagents used were purchased either from Sigma or Fisher Scientific (PA, USA) at their highest possible purity.

### Site-directed mutagenesis

pcDNA3.1-wild type (WT)-CFTR was used to generate c.3623G > A point mutation by using site-directed mutagenesis (Quickchange site-directed mutagenesis kit, Stratagene, La Jolla, CA). The primers used are:

- Forward: 5′CTGGCCCTCAGGG**GAC**CAAATGACTGTCAAAG 3′ (**GGC** > **GAC**, amino acid G > amino acid D)
- Reverse: 5′CTTTGACAGTCATTTG**GTC**CCCTGAGGGCCAG 3′

All sequences were confirmed at the Molecular Resource Center at The University of Tennessee Health Science Center.

### Cell culture and transfection

HEK293 cells were used to express WT and mutant CFTRs. The cells were cultured in DMEM/F12(1:1) (Invitrogen, Grand Island, NY, USA) medium supplemented with 1 % penicillin-streptomycin (Invitrogen) and 10 % Fetal bovine serum (Invitrogen) at 37 °C in 5 % CO_2_ incubator unless otherwise stated. Lipofectamine 2000 (Invitrogen) was used to transfect the cells following the instruction provided by the manufacturer. At 48 h post-transfection, the cells were used for western blotting or iodide efflux assay.

### Western blotting

Cells were lysed in lysis buffer (1× PBS, containing 0.2 % Triton-X-100 and protease inhibitors phenylmethylsulfonyl fluoride (1 mM), pepstatin A (1 μg/ml), leupeptin (1 μg/ml), aprotinin (1 μg/ml)) for 30 min at 4 °C. The lysates were centrifuged at 12,000 rpm for 15 min at 4 °C. The clear supernatants (the total protein levels were measured by using Bradford protein assay) were mixed with 4× sample buffer, denatured, subjected to SDS-PAGE on 5–14 % Gel (Bio-rad, Hercules, CA, USA), and transferred to PVDF membranes. The membranes were blocked with 5 % milk (in 0.1 % PBS-T) and then probed for CFTR (1:1,000 dilution) and β-actin (the loading control, 1:5,000 dilution) using the respective antibodies. The protein bands were detected using ECL™ Western blot detection reagents (GE Healthcare, Buckinghamshire, UK). The densities of bands were quantified using Image J software.

### Real-time PCR to measure CFTR mRNA levels

Total RNA was isolated from HEK293 cells transfected with WT-CFTR or G1208D-CFTR or vector using Purelink RNA Mini Kit (Thermo Fisher, Waltham, MA, USA). One microgram of total RNA was reversed to cDNA using SuperScript III Reverse Transcriptase (Invitrogen). Real-Time PCR was performed using LightCycler 480 (Roche, Indianapolis IN, USA). The primers for human CFTR were:

- Forward: 5′TTGGATCCAGTAACATACC3′
- Reverse: 5′TCAGCAGTTTCTGGATGGAATCG3′
- The primers for human GAPDH were:
- Forward: 5′TGATGACATCAAGAAGGTGG3′
- Reverse: 5′TCGTTGTCATACCAGGAAATG3′

The PCR thermocycling parameters were: 95 °C for 5 min, and 40 cycles of 95 °C for 10 s, and 60 °C for 30 s. All samples were run in triplicate and levels of CTFR messages were normalized to GAPDH.

### Iodide efflux essay

The assay was performed as previously reported [14]. Briefly, cells were grown on poly-lysine-coated 60-cm culture dishes and transiently transfected with WT-, G1208D-, or empty vector using Lipofectamine 2000. After 48 h, the cells were loaded for 60 min at room temperature with the loading buffer (136 mM NaI, 137 mM NaCl, 4.5 mM KH_2_PO_4_, 1 mM CaCl_2_, 1 mM MgCl_2_, 10 mM glucose, 5 mM HEPES, pH 7.2). Extracellular NaI was washed away thoroughly (5 times) with the efflux buffer (136 mM NaNO_3_ replacing 136 mM NaI in the loading buffer) and cells were equilibrated for 1 min in a final 1-ml aliquot of the efflux buffer. The first four aliquots were used to establish a stable baseline in efflux buffer alone. PKA activating agonists cocktail (10 μM forskolin, 200 μM CPT-cAMP, and 100 μM IBMX) was added to the efflux buffer and samples were collected every minute for 6 times in the continued presence of agonists. The iodide concentration of each sample was determined using an iodide-sensitive electrode (Orion Research Inc., Beverly, MA, USA) and the efflux rates were reported as nano-moles (nmol)/min. After experiments, the cells were lysed and the total protein concentrations measured. The maximal iodide efflux rates of samples were normalized to their total protein concentrations of cell lysates.

### Statistical analysis

The data were presented as Mean ± S.E.M. Statistical analyses were performed using Student’s *t*-test and *P* values < 0.05 were considered significant.

## Results

### Clinical case summary

The patient was identified after positive newborn screening by the state of Tennessee. The African-American infant was a product of a twin gestation born preterm at 31 weeks. The newborn screening result of this child was positive, with initial elevated immunoreactive trypsinogen of 148 ng/ml and repeat at 3 weeks of age again elevated at 123 ng/ml. At 2 months old, the sweat chloride levels were 51, 45 mmol/L and pancreatic elastase level was low at 116 μg/g (pancreatic insufficiency). Based on these lab testing results and poor weight gain, the child was diagnosed with CF and management was started, including enzyme replacement therapy. The child has had poor but steady weight gain. The sweat chloride levels were 47, 46 mmol/L at 5 months of age and 54, 48 mmol/L at 29 months of age. Stool elastase testing was >500 μg/g at 25 months of age, and pancreatic enzymes supplementation was stopped. Chest radiograph showed normal findings. There have been no admissions for pulmonary exacerbations, and throat cultures have shown growth of only gram-positive organisms. At 35 months old, the child’s height and weight were at the 5th and below the 3rd percentile, respectively. DNA sequencing test revealed that the child is heterozygous for F508del and a missense mutation, c.3623G > A.

### c.3623G > A encoding protein, G1208D-CFTR, has defect in maturation and shows impairment in channel function

**Fig. 1:**
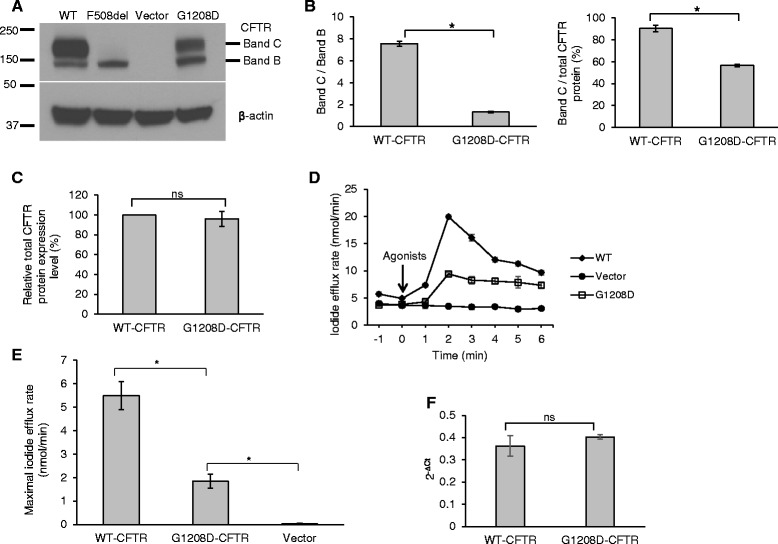
G1208D-CFTR has defect in protein maturation and shows impaired chloride channel function. **a** A representative blot showing the expression level and maturation status of G1208-CFTR, WT-CFTR, F508del-CFTR, or vector transiently expressed in HEK293 cells. Band C denotes a mature form of CFTR and Band B denotes an immature form of CFTR. **b** G1208D-CFTR has defect in protein maturation, with a mean maturation efficiency of 57 %. The data (density of the bands) were quantified from blots as represented in (**a**) using Image J software and presented as ratio of Band C to Band B (*left panel*) and ratio of Band C to total protein (B and C + B and B; *right panel*). The maturation efficiency was defined as the ratio of the mature form CFTR (density of Band C) to the total CFTR protein (density of B and C plus Band B). *P <0.05, *n* = 3. **c** The total CFTR protein expression levels of G1208D-CFTR and WT-CFTR are comparable. The densities of the bands were normalized to their loading control, β-actin, respectively. ns: not significant. *n* = 3. **d** Representative iodide efflux traces of G1208D-CFTR, WT-CFTR, or vector transiently expressed in HEK293 cells. PKA activating agonists (10 μM forskolin, 100 μM IBMX, and 200 μM cpt-cAMP) were added to activate CFTR channel function. **e** The maximal iodide efflux rate of G1208D-CFTR is 33 % of that of WT-CFTR. The data were quantified from experiments as represented in (**d**) and normalized to the total protein concentrations of cell lysates for each sample, respectively. *P <0.05, *n* = 5. **f** The levels of CFTR mRNA are similar between WT and G1208D. ns: not significant. *n* = 9

We generated c.3623G > A mutation on a pcDNA3.1-WT-CFTR background using site-directed mutagenesis. We tested the expression level and maturation status of the c.3623G > A encoding protein, p.Gly1208Asp (legacy name G1208D, which will be used in this manuscript), using western blotting. HEK293 cells were used to express the plasmids of G1208D-CFTR, WT-CFTR, F508del-CFTR, or the vector. As expected, WT-CFTR was expressed predominantly as a mature form (Band C) (Fig. 1a), with a maturation efficiency (the percentage of the mature form CFTR protein (Band C) to total CFTR protein (Band B plus Band C)) of 90 % (Fig. 1b). F508del-CFTR was expressed as an immature form (Band B) and the vector-transfected cells did not express CFTR protein (Fig. 1a). G1208D-CFTR was expressed in both mature and immature forms (Fig. 1a), with a maturation efficiency of 57 % (Fig. 1b). The total CFTR protein expression levels of G1208D and WT were comparable (Fig. 1c). We also tested the mRNA levels of G1208D-CFTR and WT-CFTR using real-time PCR and found their levels were not significantly different (Fig. 1f). Our results indicate that G1208D-CFTR has defect in intracellular processing and trafficking, leading to impaired protein maturation.

We next tested the channel function of G1208D-CFTR in HEK293 cells using iodide efflux assay. HEK293 cells transfected with WT-CFTR or vector were used as controls. As shown in Fig. 1d, upon stimulation, WT-CFTR exhibited a typical CFTR-mediated iodide efflux profile [14] whereas the vector-expressing cells did not show measureable iodide efflux. Although G1208D-CFTR showed a response, the magnitude was markedly smaller compared to WT-CFTR. The maximal iodide efflux rate of G1208D-CFTR was found to be 33 % of that of WT-CFTR (Fig. 1e). The data demonstrates that the channel function of G1208D-CFTR is impaired, which correlates with our finding that the CFTR protein has defect in maturation.

### G1208D-CFTR can be partially rescued by exposure to low temperature or using VX-809

**Fig. 2:**
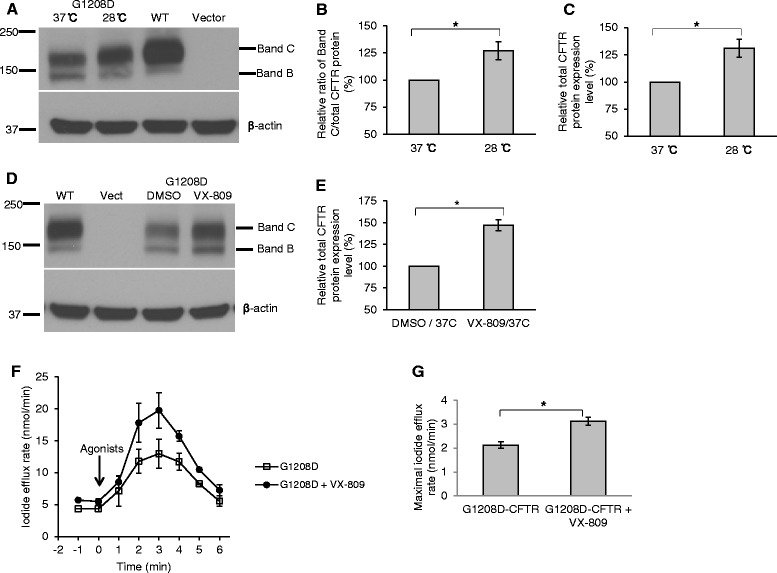
G1208D-CFTR can be partially rescued by exposure to low temperature or using VX-809. **a** A representative blot showing the expression level and maturation status of G1208D-CFTR in HEK293 cells cultured at normal condition (37 °C) or at a low temperature (28 °C). WT-CFTR- and vector-transfected HEK293 cells were cultured at 37 °C and used as controls. **b** Exposure to low temperature (28 °C) promoted the maturation efficiency (1.3-fold) of G1208D-CFTR. The data were quantified from blots as represented in (**a**) using Image J software. *P <0.05, *n* = 3. **c** Exposure to low temperature (28 °C) increased the total CFTR protein level of G1208D (1.3-fold). The data were quantified from blots as represented in (**a**) using Image J software and normalized to their loading control, β-actin, respectively. *P <0.05, *n* = 3. **d** A representative blot showing the expression level and maturation status of G1208D-CFTR in HEK293 cells treated with VX-809 (5 μM) or DMSO (solvent control for VX-809). WT-CFTR- and vector-transfected HEK293 cells were used as controls. **e** VX-809 increased the total CFTR protein level of G1208D-CFTR (1.5-fold). The data were quantified from blots as represented in (**d**) using Image J software and normalized to their loading control, β-actin, respectively. *P <0.05, *n* = 3. **f** Representative iodide efflux traces of G1208D-CFTR in HEK-293 cells treated with VX-809 (5 μM) or DMSO. PKA activating agonists (10 μM forskolin, 100 μM IBMX, and 200 μM cpt-cAMP) were used to activate CFTR channel function. **g** The maximal iodide efflux rate of G1208D-CFTR increased 1.5-fold with VX-809 treatment compared to DMSO-treated controls. The data were quantified from experiments as represented in (f) and normalized to the total protein concentrations of cell lysates for each sample, respectively. *P <0.05, *n* = 9–11

Based on the findings that G1208D-CFTR exhibited defect in processing (Fig. 1), which is a characteristics of the class II mutations (e.g., F508del), and that F508del-CFTR can be partially rescued at low temperature or using CFTR correctors [15], we continued to investigate whether G1208D-CFTR can be rescued by using a CFTR corrector VX-809 or exposure to low temperature (28 °C). To test the temperature effect, HEK293 cells were transfected with G1208D-CFTR and cultured at 37 °C or 28 °C. Cells transfected with WT-CFTR or vector was used to facilitate the characterization. We found that exposure to low temperature (28 °C) increased the total CFTR protein level (1.3-fold) and promoted the maturation of G1208D-CFTR (a 1.3-fold increase) compared to cultures at 37 °C (Fig. 2a, b, c). To test the effect of VX-809, HEK293 cells were transfected with G1208D-CFTR and treated with VX-809 or DMSO (solvent control) and cultured at 37 °C. We found that VX-809 significantly increased the total CFTR protein level (1.5-fold) of G1208D-CFTR (Fig. 2d, e) and slightly promoted its maturation (a 1.1-fold increase). Of note, we did not find an additive or synergetic effect with the use of VX-809 and exposure at low temperature.

We then tested whether VX-809 potentiates the G1208D-CFTR channel function in HEK293 cells using iodide efflux assay. We found that VX-809 did augment the channel function of G1208D-CFTR, with a 1.5-fold increase in the maximal iodide efflux rate compared to DMSO control (Fig. 2f, g).

## Discussion

Newborn screening programs have facilitated the identification of rare or unique CFTR mutations in populations such as Africa-Americans where classical CF presentations are uncommon [16, 17]. Many of these rare mutations have not been well characterized with regard to CFTR channel function and there is a risk that mutations that potentially respond to CFTR-modifying therapies may not be identified in these populations.

Our biochemical findings demonstrate that G1208D-CFTR has a moderate processing defect, characterized by features of impaired CFTR protein maturation and channel function, as well as sensitivity to temperature and responsiveness to correction with VX-809. Although it is well known that variables in genetic background (e.g. modifier genes) and environment factors also affect CF disease manifestations [18], our finding that G1208D-CFTR has residual CFTR function (33 % of normal CFTR) correlates well with the mild disease phenotype in this patient, with preserved pancreatic function and intermediate sweat chloride levels, when paired with a classical CFTR mutation like F508del.

Our patient is still too young to evaluate the effect of the mutation on lung damage but it has been known that patients with residual CFTR channel function can still develop bronchiectasis with chronic sino-pulmonary infections and other CF-related diseases (e.g., recurrent pancreatitis, CF-related diabetes, male infertility) when they age [8]. Given that the two mutations this patient has, F508del and c.3623G > A, are both responsive to VX-809, we speculate that VX-809 and similar CFTR modulators could be beneficial for his therapy.

## Conclusions

We report the clinical features of a pediatric CF patient heterozygous for F508del and a novel missense c.3623G > A mutation. We characterized c.3623G > A mutation at the molecular level using cell culture models and found that the mutant protein has a moderate processing defect and an impaired channel function, which correlates with the mild disease phenotype present in this patient. Our study suggests that c.3623G > A mutation is a mild CF-causing mutation. We also found that the mutant protein can be partially rescued by using a CFTR corrector VX-809 or exposed at a low temperature. Our findings expand our knowledge of rare CFTR mutations, demonstrate that VX-809 restores function to other rare mutations in addition to F508del, and may have clinical implications for possible therapeutic interventions for this and other patients with c.3623G > A mutation.
